# Expression of MAPK and PI3K/AKT/mTOR Proteins according to the Chronic Liver Disease Etiology in Hepatocellular Carcinoma

**DOI:** 10.1155/2020/4609360

**Published:** 2020-10-29

**Authors:** Paulo H. C. Diniz, Serena D. C. Silva, Paula V. T. Vidigal, Marcelo A. P. Xavier, Cristiano X. Lima, Luciana C. Faria, Teresa C. A. Ferrari

**Affiliations:** ^1^Departamento de Clínica Médica, Faculdade de Medicina, Universidade Federal de Minas Gerais, Belo Horizonte, MG, Brazil; ^2^Departamento de Anatomia Patológica e Medicina Legal, Faculdade de Medicina, Universidade Federal de Minas Gerais, Belo Horizonte, MG, Brazil; ^3^Departamento de Cirurgia, Faculdade de Medicina, Universidade Federal de Minas Gerais, Belo Horizonte, MG, Brazil

## Abstract

**Aims:**

Chronic liver disease (CLD) of different etiologies leads to hepatocellular carcinoma (HCC) by multiple mechanisms that may be translated into clinicopathological differences. We evaluated the tissue expression of the MAPK and PI3K/Akt/mTOR pathway proteins and their association with long-term outcome and other parameters, according to the etiology of the CLD, in HCC patients.

**Methods:**

Clinicopathological data from 80 patients who underwent orthotopic liver transplantation for HCC treatment in a Brazilian referral center were compared according to CLD etiology. Event (tumor recurrence or death from any cause) occurrence and event-free survival (EFS) were analyzed. Pathway protein expression was assessed by immunohistochemistry (IHQ) in both tumor and underlying cirrhosis and by RT-PCR in tumor tissue.

**Results:**

Strong expression (SE) of KRAS was more frequent in tumors arising from viral (26.8%) than the nonviral group of liver disease (7.7%, *p*=0.024) and also than cirrhotic parenchyma (0%, *p*=0.004). SE of PI3K was more frequent in tumor than in cirrhosis (*p*=0.048, *p* < 0.01), without differences in its tumor expression among etiologic groups (*p*=0.111). mRNA of ERK, PI3K, and BRAF was expressed in the tumor, without differences between CLD etiologies, and there was no association with IHQ findings. Older age and microvascular invasion (MIV) were the only parameters independently associated with the event. MIV was also associated with shorter EFS.

**Conclusions:**

Hepatitis B and C virus can lead to HCC by different mechanisms compared with nonviral hepatopathy. KRAS and PI3K may have a role in carcinogenesis. The prognostic and therapeutic implications need to be investigated.

## 1. Introduction

Hepatocellular carcinoma (HCC), whose incidence has risen over the last 20 years in many countries, still has a dismal prognosis and nowadays ranks the fourth as the leading cause of cancer death worldwide [1]. This complex and heterogeneous malignancy is caused by chronic liver disease (CLD) of different etiologies, which show a large geographic variation: hepatitis B virus (HBV), hepatitis C virus (HCV), excessive alcohol consumption, autoimmune disorders, nonalcoholic fatty liver disease (NAFLD), inherited metabolic disorders, and other nonidentified etiologies, called cryptogenic [2].

Hepatocarcinogenesis is poorly understood but is recognized as a multistep process in which several pathways can cooperate [3]. Among them, regulators of cell proliferation and survival stand out [4]. In this context, different causes of the underlying CLD may induce diverse oncogenic mechanisms and some of these pathways can be related to specific etiologies [5, 6].

The mitogen-activated protein kinase (MAPK) cascade and the PI3K/Akt/mTOR are the best characterized and more frequently activated intracellular pathways in HCC, suggesting a possible role in its pathogenesis [4, 7]. The first one, activated in up to 51% of HCC cases, seems to have a universal expression in advanced stages [6]. The mTOR pathway, with activation changes ranging from 15 to 41% in this cancer, [8–11], is associated with aggressive tumor behavior and decreased survival [12].

Through a sequence of phosphorylation events in the MAPK (RAS/RAF/MEK/ERK) and PI3K/Akt/mTOR pathways, downstream signaling may activate transcription factors, which modify the expression of proteins involved in important cellular processes, such as proliferation, differentiation, apoptosis, cell cycle progression, tumorigenesis, tumor growth, and angiogenesis [13, 14]. Originally, modeled as linear signaling conduits activated by different stimuli, these important pathways could intersect to regulate each other and coregulate other functions [15].

Despite the frequent activation of these intracellular pathways in HCC, mutations related to the expression of these proteins are rare-identified in <2% in some studies, and additional mechanisms of activation remain to be identified [3, 16]. Moreover, variations associated with the etiology of the underlying CLD and among different regions of the world need to be investigated [17].

Available evidence suggests that diverse and complex mechanisms are involved in hepatocarcinogenesis. The etiology of the CLD may have a role in this process, but the molecular aspects are poorly understood. Recognizing how it occurs and how it can be translated into clinicopathological and prognostic differences could contribute to improving patient care. In this study, we evaluated the expression MAPK pathway and PI3K/Akt/mTOR proteins in HCC and investigated their association with clinical and histopathological parameters and long-term outcomes, according to the etiology of the underlying CLD.

## 2. Methods

### 2.1. Cohort

From 1998 to 2015, 80 of the 156 patients who underwent orthotopic liver transplantation (OLT) for HCC treatment at Hospital das Clinicas, Universidade Federal de Minas Gerais, a referral center in Brazil, were randomly selected according to the most frequent CLD etiologies. All patients had archived formalin-fixed, paraffin-embedded (FFPE) tissue of the explanted liver.

Clinical pre-OLT data of the patients and histopathologic parameters of the explanted liver were retrospectively collected from the medical records. In this study, an event was considered as tumor recurrence or death from any cause. If the patient presented with both occurrences, only the first event was counted. Event-free survival (EFS) was defined as the time interval between the OLT date and the event occurrence or the follow-up period ending (December 20, 2017). If necessary, the patient or his family was assessed by phone.

This study was approved by the local Ethics Committee (CAAE-44573615.7.0000.5149), and written informed consent was obtained from the patients or their relatives.

### 2.2. Immunohistochemistry

Tissue microarrays, a technique in which different samples are ordered in the same paraffin blocks, were constructed as previously described [18]. As a representative, two 0.6 mm cores of FFPE tumor samples were taken from the explanted liver of each patient, with adjacent cirrhosis whenever it was possible and placed in blocks according to the etiologic group of CLD.

Four-micrometer paraffin sections were dried, deparaffinized, and dehydrated. After antigen retrieval, the next steps were performed using a Novolink Polymer Detection Systems kit (Leica Biosystems, UK), according to the manufacturer's recommendations.

The primary antibodies are shown in Table S1 in Supplementary Materials. We used a biotinylated secondary antibody and Poly-HRP (horseradish peroxidase) conjugated to anti-rabbit-IgG. The reactions were revealed by applying 3,3′-diaminobenzidine, and the sections were counterstained with hematoxylin (Fisher Scientific, USA).

Evaluation of tissue array slides on an optical microscope was performed by two experienced pathologists, who were blinded to the details of the patients, and the results were determined based on their agreement. The used scoring system, integrating the intensity and extent of staining for every sample and every antibody, was adapted from a previous publication [19]. The intensity of staining was scored as 0 (negative), 1-2 (weak), or 3-4 (strong). The extent of staining was scored according to the number of positive tumor cells: 0 (negative), 1 (1–25%), 2 (26–50%), 3 (51–75%), and 4 (76–100%). The final score of each sample and each protein, separately for tumor and adjacent cirrhosis, was assessed by the product of the intensity and extent of staining, and the average of each marker and tumor was determined. Thereafter, each case was finally categorized into weak expression (score 0–8) and strong expression (SE) (score 9–16). A negative control, in which primary antibodies were omitted, was included in all antibodies. The positive control was assessed as indicated in the user manual of each primary antibody.

### 2.3. Real-Time PCR

According to the manufacturer's recommendations, we extracted total cellular RNA from tumor section regions of FFPE blocks using the RNeasy FFPE Kit (QIAGEN, 73504, Germany). cDNAs were generated using the Master Mix kit (Invitrogen, 11766-050, USA). Quantitative real-time polymerase chain reactions (RT-PCRs) were conducted with SYBR Green PCR Supermix (Bio-Rad, USA), using PCR primers (Ludwig Biotecnologia, Brazil) on a Fast Real-Time PCR System (Applied Biosystems, USA), described in Table S2 in Supplementary Materials. Melting curve analysis evaluated the primer specificities. The results—expressed in cycle threshold (Ct)—were normalized to the level of glyceraldehyde 3-phosphate dehydrogenase (GAPDH). Three technical replicates were used per sample. Relative mRNA expression was determined by the comparative Ct method using Bio-Rad software (Bio-Rad, USA).

### 2.4. Statistical Analysis

Descriptive statistics were used to summarize the data. A normality test (Shapiro-Wilk) was performed for each continuous variable. RT-PCR data represent at least three independent experiments. For comparison between groups, we used the Chi-square or Fisher's exact test, if categorical variables, and we used the Mann–Whitney *U* test or the Kruskal-Wallis test, if continuous data. In multiple comparisons, Bonferroni correction was applied.

According to the *Z* test, the study required 36 patients in each group (viral versus nonviral etiology) to have 80% power to detect at least 25% of the difference in SE of KRAS in immunohistochemistry (IHQ), assuming a significance level of 0.05.

A multivariate Poisson regression model with covariance structure was performed to identify the characteristics independently associated with the event, and COX regression analysis was performed to evaluate EFS. Variables associated with the endpoint in univariate analysis (*p* < 0.20) were included in the multivariate model. Statistical significance was assumed at *p* < 0.05. We used SPSS software, version 20 (SPSS, Chicago, IL).

## 3. Results and Discussion

### 3.1. Clinicopathological Parameters and Clinical Outcomes according to CLD Etiology

Patients' demographic, clinical, laboratory, and histopathological characteristics according to the CLD etiology are summarized in Table 1. The groups were well balanced. Considering the morphological variables, there were no significant differences among the etiologic groups.

Regarding clinical outcomes (Table 2), no differences among the etiologic groups were demonstrated. The median follow-up period was 63 months (range, 1–104.5 months). The event rate was 38% (27 recurrences or deaths from any cause among the 71 patients analyzed). In nine patients, event occurrence could not be assessed (lost to follow-up). HCC recurrence was diagnosed in five of the 71 individuals (7%), although it may also have occurred in patients who died. The median EFS was 75.4 months (range, 8.5–105.2 months), without difference among the etiologic groups.

### 3.2. Expression of MAPK and PI3K/AKT/mTOR Pathway Proteins according to CLD Etiology

**Figure 1:**
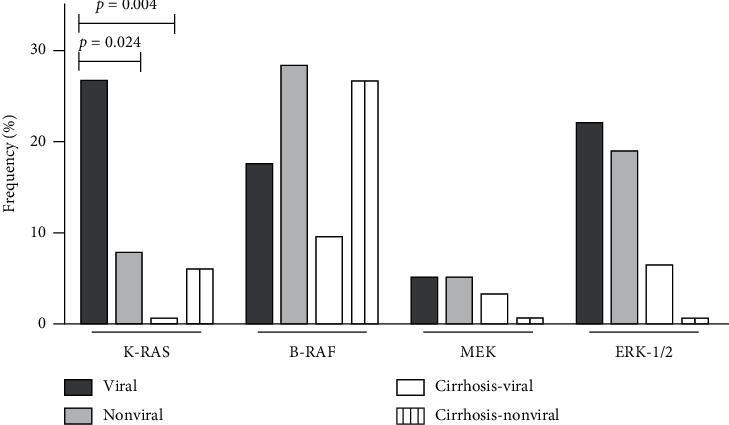
Frequency of strong expression of MAPK pathway proteins in hepatocellular carcinoma patients according to the etiology of the underlying chronic liver disease in tumor and adjacent cirrhosis.

**Figure 2:**
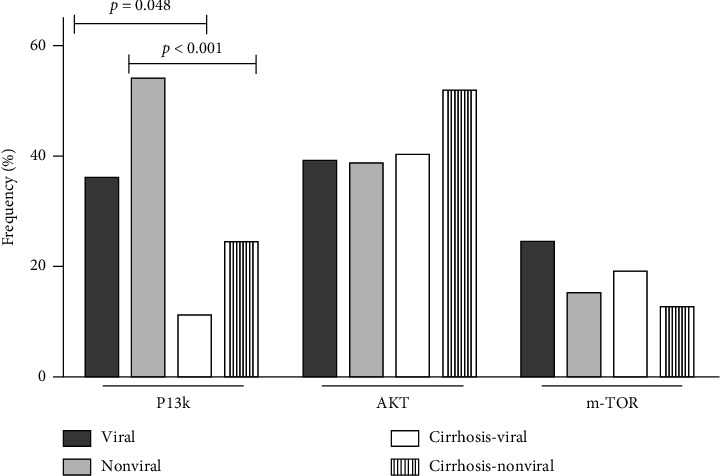
Frequency of strong expression of PI3K/AKT/mTOR pathway proteins in hepatocellular carcinoma patients according to the etiology of underlying chronic liver disease in tumor and adjacent cirrhosis.

The results of the analysis of the pathway proteins SE according to the etiologic group, in both tumor and adjacent cirrhosis, are described in Tables 3 and 4 and summarized in Figures 1 and 2. The representative immunohistochemical maps are shown in Figures S1 and S2 in Supplementary Materials. In the viral group, 11 of 41 patients (26.8%) had SE of KRAS in the tumor, compared to 0/33 (0%) in cirrhosis (*p*=0.008) and 3/39 (7.7%) in nonviral group HCC (*p*=0.024). There was no difference between KRAS SE in the tumor and cirrhosis in the nonviral group (*p*=1.000). Regarding SE of PI3K, we found a difference between tumor and adjacent cirrhosis: 14/39 (35.9%) versus 3/27 (11.1%) in the viral etiologic group (*p*=0.048), and 21/30 (53.8%) versus 8/33 (24.2%) in the nonviral group (*p* < 0.001), but no difference was observed when comparing PI3K SE in the tumor between the etiologic groups (*p*=0.111). SE of the other proteins was often present, but differences regarding the expression profile, considering tumor and adjacent cirrhosis as well as the etiologic groups, were not demonstrated.

**Figure 3:**
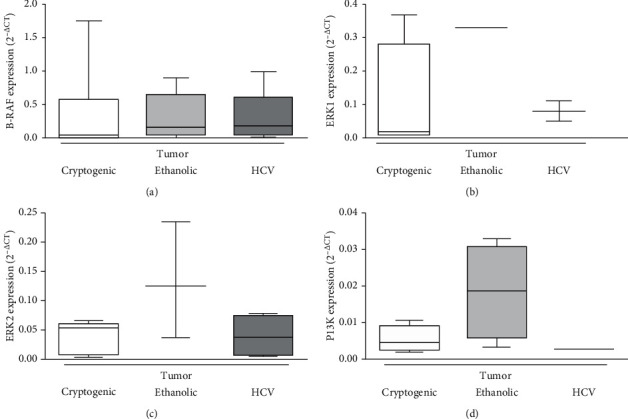
Relative RNA expression of some pathway proteins in tumor tissue according to the etiology of the underlying chronic liver disease. The data are normalized for the level of GAPDH and expressed as 2^−ΔCt^. ΔCt refers to the difference between Ct of GAPDH and Ct of primer analyzed per sample. The results are expressed as median (interquartile range), three technical replicates per sample. (a) BRAF 0.423 (0.003–0.573), *n* = 8/8; 0.222 (0.052–0.722), *n* = 7/8; 0.183 (0.043–0.604), *n* = 8/10; *p*=0.25. (b) ERK1 0.182 (0.008–0.281), *n* = 4/8; median NA, *n* = 3/8; 1.041 (0.064–18.480), *n* = 4/10; p = =0.08. (c) ERK2 0.033 (0.006–0.633), *n* = 4/8; 0.125 (0.037–NA), *n* = 3/8; 0.375 (0.006–0.744), *n* = 4/10; *p*=0.258. (d) PI3K 0.007 (0.002–0.383), *n* = 4/8; 0.188 (0.006–0.308), *n* = 4/8; median NA, *n* = 2/10; *p*=0.480. All data refer to cryptogenic, ethanolic, and HCV etiologic groups, respectively. Comparisons were performed among the three groups (Kruskal–Wallis test) or between two groups (Mann–Whitney *U* test), and no differences were detected. It was impossible to extract RNA from the hepatitis B virus etiology and data are not shown. AKT and mTOR were also tested, but the relative RNA expression was not specifically labeled, and the data could not be shown. GAPDH, glyceraldehyde 3-phosphate dehydrogenase; HCV, hepatitis C virus, Ct, cycle threshold; CLD, chronic liver disease; NA, not available.

To analyze quantitatively the mRNA expression of the pathway proteins, RT-PCR was performed in tumor tissue and the data are shown in Figure 3. The expression of mRNA of BRAF, PI3K, ERK1, and ERK2 was demonstrated, but differences among the etiologic groups were not found, even when the etiologies were categorized into viral and nonviral groups. Only in a limited number of samples of all etiologic groups, it was possible to obtain specific mRNA amplification. Proteins could not be extracted from FFPE tumor samples of the HBV etiology, and then analyses were not performed. The samples were also tested for AKT and mTOR primers, but no specific amplification could be demonstrated.

### 3.3. Relationship between Clinicopathological Parameters and MAPK and PI3K/AKT/mTOR Pathway Protein Expression

The associations between clinical and pathological parameters with pathway protein expression, here defined as the endpoint, were evaluated. For each protein, some variables met the criteria to be included in the multivariate analysis: etiology of CLD, age, Child-Pugh score (CHILD), number of nodules, and AFP for KRAS expression; histologic grade and AFP for BRAF; gender, Model for End-Stage Liver Disease (MELD) score, CHILD, histologic grade, and AFP for MEK; CHILD and size of biggest nodule for ERK1/2; AFP for PI3K; MIV and AFP for AKT; and CHILD and MIV for mTOR. However, as shown in the final model, for each protein expression, the only independent association occurred between the number of nodules (up to 3) and SE of KRAS (odds ratio (OR), 1.32; 95% confidence interval (CI), 1.15–1.52; *p* < 0.01).

### 3.4. Influence of Pathway Proteins Expression and Clinicopathological Parameters on Event Occurrence and EFS

Finally, we evaluated the influence of pathway proteins and clinicopathological parameters on event occurrence and EFS. Only MIV and age were selected to be included in the multivariate analysis. In the final model, both MIV (OR, 13.46; 95% CI, 3.56–50.9; *p* < 0.01) and older age (OR, 1.09; 95% CI, 1.01–1.20; *p*=0.04) were independently associated with event occurrence. The MAPK and PI3K/AKT/mTOR protein expression was not associated with this outcome in the univariate analysis and neither entered the multivariate model.

Regarding EFS, three variables entered the multivariate analysis: age, number of nodules, and MIV. However, the presence of MIV was the only parameter independently associated with this endpoint (hazard ratio (HR), 4.09; 95% CI, 1.78–9.41; *p* < 0.01), indicating a risk 4.09 times higher of recurrence or death in the follow-up period for patients with MIV. The expression of any pathway protein did not associate with EFS.

## 4. Discussion

In this study, SE of MAPK and PI3K/AKT/mTOR proteins were frequent in HCC samples from patients who underwent OLT as HCC treatment, indicating activation of these signalizing pathways in such population. Moreover, KRAS expression was stronger in tumors that arose from CLD caused by HBV or HCV than in those of nonviral etiology or in cirrhotic parenchyma without tumor. PI3K was strongly expressed in tumors of both etiologic groups, but not in cirrhosis.

HCC is a complex disease, and multiple barriers have been recognized in hepatocarcinogenesis knowledge [20]. Cancer heterogeneity is particularly challenging. As differences might occur even within a single tumor nodule, the analysis performed in a small region of the tumor might not reflect the entire molecular profile of HCC, and failure to identify molecular biomarkers may occur [5].

Many genetic and molecular alterations vary according to the cancer stage [21]. For instance, TERT promoter mutation is an early step, while the acquisition of genomic diversity appears to be a late event in liver carcinogenesis [22]. Our study included only HCC patients who underwent OLT, whose disease is supposed to be limited and may not represent the molecular findings of the later stages. Despite this limitation, selecting these patients guarantees the availability of the tumor specimen for analysis, as pathological proof is still not mandatory for HCC diagnosis [3].

HCC molecular profile may also vary according to the etiology of the underlying CLD and patients' genetic background, which could be translated into geographic differences [16]. Therefore, studies evaluating molecular differences among HCC-related CLD etiologies are very important in this scenario and have never been conducted in our setting. Moreover, the correlation of molecular characteristics with clinical and pathologic features, as well as relevant clinic outcomes, could contribute to distinguishing mutation involved in cancer biology from passenger mutations [16].

In the last years, many attempts have been made to identify prognostic and predictive parameters in HCC [23]. Some of them were evaluated in this study. Alpha-fetoprotein (AFP), a biomarker that may also predict recurrence risk after transplantation, and the severity of the underlying CLD, estimated from the CHILD and MELD scores, were also assessed in this study, but no association with clinical outcome was demonstrated [24, 25]. Older age was independently associated with HCC-recurrence in our study; however, this association has been ascribed to confounding factors [26]. Therefore, the clinical relevance of this finding is unclear.

Histologic grading is expected to reflect the tumor's biological behavior in HCC, and MIV is considered a reliable predictor of tumor recurrence [27, 28]. We found high histologic grade in 28.7% of the patients and MIV in 36.7%, indicating aggressive histology in a significant proportion of our population. However, only MIV was shown to be independently associated with HCC recurrence.

Our study also showed a more frequent expression of intracellular MAPK and PI3K/AKT/mTOR proteins in tumors than in underlying cirrhosis, suggesting a critical role in HCC development. Aberrant upstream signals originated from proteins extrinsic to the pathway, such as EGFR (epithelial growth factor receptor), and oncogenic viral proteins, such as HBV X protein and HCV core protein, have been recognized [20, 29–32]. Moreover, activation of PI3K/AKT/mTOR can be a result of reduced activity of PTEN, its negative regulator, or EGF upregulation [33].

Evidence suggests activation of the mTOR pathway in poorly differentiated tumors, in the presence of vascular invasion, and in association with other poor prognostic features [33]. Likewise, the MAPK pathway activation is associated with a worse prognosis [34]. In our study, the multivariate analyses showed an independent association between the number of nodules (up to three) and SE of KRAS, but the relevance of this finding is not clear. No other association between protein expression and clinicopathological parameters or long-term outcome was demonstrated.

Despite both pathways were more frequently activated in HCC than in cirrhosis, our results showed that the frequency of protein expression did not follow a predictable sequence. For example, the expression of MEK-1 was less common than its downstream protein (ERK-1/2), and less than BRAF, its upstream step. It highlights the complexity of pathways, in which upstream and downstream proteins do not necessarily follow a linear activation sequence, as originally modeled. In addition, the pathways cross-talk extensively and regulate each other [15]. Furthermore, other effectors with potential clinical relevance have been identified, like PI3K, which can trigger a downstream activation, not dependent on AKT [35]. This suggests a role for additional inputs in each pathway, which can influence the protein expression. However, it is important to mention that in the experiments we did not investigate the phosphorylation of proteins, like AKT, which could be activated with no increase in its expression.

Because of the limited ability of IHQ in measuring protein expression, the bias in favoring the selection of “representative” tumor areas, and certain subjectivity in the quantification, we performed RT-PCR, which reflects the average mRNA expression of the sample [36]. In the present study, we showed the expression of mRNA of many genes (BRAF, ERK1, ERK2, and PI3K), but with no differences among the etiologic groups. Others, like AKT and mTOR, did not show specific amplification, probably due to lack of RNA preservation or issues related to the primers. That is the reason why KRAS and MEK-1 were not assessed. In fact, gene expression analyses of RNA isolated from FFPE tissues are challenging [37].

Notably, in our study, the mRNA levels, measured by RT-PCR, did not correspond necessarily to the levels of protein expressed, evaluated by IHQ. It may reflect posttranslational modifications or the increased dynamic range of RT-PCR as compared to IHC [37]. Many complicated and varied posttranscriptional mechanisms involved in turning mRNA into protein are not yet sufficiently well-defined and it is not feasible to estimate protein concentration from mRNA; proteins may differ substantially in their in vivo half-lives; and/or experimental errors may have limited the results [38].

As other limitations, our study was retrospective and performed in a single-center, not reflecting the geographic and molecular diversity of our population. However, this is the first cohort in which molecular, clinical, pathological, and long-term outcomes in a poor-income country were evaluated. The reduced representability of the HBV group is also an important issue, considering that this infection itself can be a poor prognostic factor [39]. However, its prevalence has decreased in Brazil, favored by vaccination and the easier access to antiretroviral treatment [40]. Finally, NAFLD, only recently recognized as an HCC etiology, was not considered as a separated category but probably included in the cryptogenic group [41].

In summary, our study showed increased activity of MAPK and PI3K/ATK/mTOR pathways in HCC patients who underwent OLT. SE of KRAS was more frequent in viral-related HCC. The expression of PI3K was most often strongly expressed in tumor than in cirrhosis. These findings suggest that hepatitis viruses can lead to HCC by different mechanisms compared to CLD of nonviral etiology, and KRAS and PI3K could have a role in carcinogenesis. As there were no differences in clinical outcomes, further studies are needed to investigate possible implications.

## Figures and Tables

**Table 1:** Demographic, clinical, and histopathological parameters of HCC patients according to CLD etiology.

Characteristics	All etiologies	Viral group (*n* = 41)		Nonviral group (*n* = 39)		*p* value
		HBV	HCV	Alcohol	Cryptogenic	
Number	80 (100.0)	10 (12.5)	31 (38.8)	20 (25.5)	19 (23.7)	NA

Age (years)	58.0 (52.0–64.0)	56.0 (38.8–60.4)	56.0 (51.0–62.0)	58.5 (52.0–63.7)	63.0^a^ (57.0–66.8)	0.023^b^

Gender						
F	20 (25.0)	1 (10.0)	12 (38.7)^a^	1 (5.0)	6 (31.6)	0.021^b^
M	60 (75.5)	9 (90.0)	19 (61.3)	19 (95.0)^a^	13 (68.4)	—
MELD	20 (16–24)	21(20–28)	20 (14–26)	20 (17–23)	20 (17–22)	0.760^b^

CHILD						
A	29/67 (43.2)	6/8 (75.0)	13/25 (52.0)	5/19 (26.3)	5/15 (33.3)	0.205^**c**^
B	19/67 (28.4)	1/8 (12.5)	8/25 (32.0)	6/19 (31.6)	4/15 (26.7)	—
C	19/67 (28.4)	1/8 (12.5)	4/25 (16.0)	8/19 (42.1)	6/15 (40.0)	—
AFP (ng/mL)	14.7 (5.87–72.4)	14.1 (3.17–585.2)	25.1 (8.2–73.6)	14.2 (5.3–64.3)	7.4 (4.4–94.3)	0.421^b^

No. of nodules						
≤3	58/75 (77.3)	8/9 (88.9)	23/30 (76.7)	16 (80)	11/16 (68.8)	0.751^c^
>3	17/75 (22.7)	1/9 (11.1)	7/30 (23.3)	4 (20)	5/16 (31.3)	—
Size of biggest nodule (cm)	2.8 (2.0–3.5)	2.8 (1.7–5.2)	2.5 (2.1–3.5)	2.6 (2.0–3.4)	3.25 (2.5–5.0)	0.411^b^

Histologic grade						
Low	52/73 (71.2)	8 (80.0)	17/28 (60.6)	12/18 (66.7)	15/17 (88.2)	0.221^c^
High	21/73 (28.7)	2 (20.0)	11/28 (39.3)	6/18 (33.3)	2/17 (11.8)	—

MIV						
No	45/71 (63.3)	9 (90)	14/27 (51.9)	11/17 (64.7)	11/17 (64.7)	0.166^c^
Yes	26/71 (36.7)	1 (10)	13/27 (48.1)	6/17 (35.3)	6/17 (35.3)	—

Data are expressed as absolute numbers (percentage) and median (interquartile range). Number of patients with the characteristic/number for whom the information was available. HCC, hepatocellular carcinoma; CLD, chronic liver disease; HBV, hepatitis B virus; HCV, hepatitis C virus; NA, not applicable; F, female; M, male; MELD, Model for End-Stage Liver Disease; CHILD, Child-Pugh classification; AFP, alpha-fetoprotein; MIV, microvascular invasion. ^a^Statistically significant difference. ^b^Kruskal–Wallis test. ^c^Fisher's exact test.

**Table 2:** Clinical outcome in HCC patients according to CLD etiology.

Criteria	All etiologies	Viral group		Nonviral group		*p* value
		HBV	HCV	Alcohol	Cryptogenic	
Recurrence	5/71 (7.0)	1/10 (10.0)	3/27 (11.1)	1/20 (5.0)	0/14 (0)	0.635
Event	27/71 (38.0)	4/10 (40.0)	11/27 (40.7)	5/20 (25.0)	7/14 (50.0)	0.509
EFS (months)	74.5 (8.5–105.2)	41.0 (2.5–99.0)	87.0 (39.0–120.2)	75.5 (38.5–100.7)	34.0 (0.0 – 109.2)	0.470

Data are expressed as absolute numbers (percentage) and median (interquartile range). Number of patients with the characteristic/number for whom the information was available. HCC, hepatocellular carcinoma; CLD, chronic liver disease; HBV, hepatitis B virus; HCV, hepatitis C virus; EFS, event-free survival. The event was defined as recurrence or death from any cause since liver transplantation. Fisher's exact test.

**Table 3:** Strong expression of MAPK pathway proteins in HCC patients in both tumor and adjacent cirrhosis according to CLD etiology.

Etiology	KRAS			BRAF			MEK			ERK-1/2		
	Tumor *n* (%)	Cirrhosis *n* (%)	*p* value	Tumor *n* (%)	Cirrhosis *n* (%)	*p* value	Tumor *n* (%)	Cirrhosis *n* (%)	*p* value	Tumor *n* (%)	Cirrhosis *n* (%)	*p* value
Viral	11/41 (26.8)	0/33 (0.0)	0.004^b^	7/40 (17.5)	3/32 (9.4)	0.755^b^	2/41 (4.9)	1/32 (3.1)	1.000^b^	7/32 (21.9)	2/32 (6.3)	0.150^b^

Nonviral	3/39 (7.7)	2/34 (5.9)	1.000^b^	11/39 (28.2)	8/30 (26.6)	1.000^b^	2/39 (5.1)	0/32 (0.0%)	0.563^b^	6/32 (18.8)	0/30 (0.0)	0.390^b^

*p* value	0.024^a^	0.049^b^	—	0.257^a^	0.075^a^	—	1.000^b^	1.000^b^	—	0.756^a^	0.492^b^	—

Data are expressed as the absolute number/total of samples analyzed (percentage). MAPK, mitogen-activated protein kinases; HCC, hepatocellular carcinoma; CLD, chronic liver disease. ^a^Chi-square test. ^b^Fisher's exact test.

**Table 4:** Strong expression of PI3K/AKT/mTOR pathway proteins in HCC patients both in tumor and adjacent cirrhosis according to CLD etiology.

Etiology	PI3K		AKT				mTOR	
	Tumor *n* (%)	Cirrhosis *n* (%)	*p* value	Tumor *n* (%)	Cirrhosis *n* (%)	*p* value	Tumor *n* (%)	Cirrhosis *n* (%)	*p* value
Viral	14/39 (35.9)	3/27 (11.1)	0.048^b^	16/41 (39.0)	14/35 (40.0)	1.000^b^	10/41 (24.4)<	6/32 (18.8)	0.769^b^
Nonviral	21/30 (53.8)	8/33 (24.2)	<0.001^b^	15/39 (38.5)	15/29 (51.7)	0.399^b^	6/39 (15.4)	4/32 (12.5)	1.000^b^
*p* value	0.111^a^	0.315^b^	—	0.959^a^	0.348^a^	—	0.314^a^	0.491^a^	—

Data are expressed as the absolute number/total of samples analyzed (percentage). HCC, hepatocellular carcinoma; CLD, chronic liver disease. ^a^Chi-square test. ^b^Fisher's exact test.

## Data Availability

The data that support the findings of this study are available from the corresponding author on reasonable request, since we respect the Ethics Committee to protect patient confidentiality.

## Abbreviations

CLD: Chronic liver disease
HCC: Hepatocellular carcinoma
OLT: Orthotopic liver transplantation
EFS: Event-free survival
IHQ: Immunohistochemistry
SE: Strong expression
MIV: Microvascular invasion
HBV: Hepatitis B virus
HCV: Hepatitis C virus
NAFLD: Nonalcoholic fatty liver disease
CHILD: Child-Pugh score
MELD: Model for End-Stage Liver Disease
RT-PCR: Real-time polymerase chain reaction (RT-PCR)
AFP: Alpha-fetoprotein
FFPE: Formalin-fixed paraffin-embedded
CT: Cycle threshold
GAPDH: Glyceraldehyde 3-phosphate dehydrogenase
HR: Hazard ratio.

## Supplementary Materials

Supplementary Materials Table S1: list of primary antibodies, dilutions, buffer, and incubation times used in IHC. Table S2: sequences of primers used in the study. Figure S1 (A–H): representative staining patterns. Figure S2 (A–F): representative staining patterns.
