# Supplementary material for: Expression of MAPK and PI3K/AKT/mTOR Proteins according to the Chronic Liver Disease Etiology in Hepatocellular Carcinoma
Source: J Oncol. 2020 Oct 29;2020:4609360. doi: 10.1155/2020/4609360 (PMC7644337; doi:10.1155/2020/4609360)
Supplement: Supplementary Materials — Table S1: list of primary antibodies, dilutions, buffer, and incubation times used in IHC. Table S2: sequences of primers used in the study. Figure S1 (A–H): representative staining patterns. Figure S2 (A–F): representative staining patterns. [file 4609360.f1.docx]

Supplementary material

| **Table S1: List of primary antibodies, dilutions, buffer and incubation times used in IHC** | | | |
| --- | --- | --- | --- |
| **Primary Ab** | **Specifications** | **Diluition** | **Incubation time** |
| KRAS | ABCAM, ab180772, United Kingdom | 1:100 | Overnight |
| BRAF | Santa Cruz Biotechnology, sc-900, USA | 1:200 | Overnight |
| MEK-1 | ABCAM, ab32091, United Kingdom | 1:100 | 2 hours |
| ERK1/2 | Santa Cruz Biotechnology, sc-135900, USA | 1:250 | 2 hours |
| PI3K | ABCAM, ab86714, United Kingdom | 1:200 | 2 hours |
| AKT | ABCAM, ab8805, United Kingdom | 1:500 | 30 minutes |
| mTOR | ABCAM, ab32028, United Kingdom | 1:100 | 2 hours |

Abbreviations: IHQ, immunohistochemistry; ab, antibody; Phosphate-buffered saline (PBS) was used as diluent.

| **Table S2: Sequences of primers used in the study** | |
| --- | --- |
| **Identification** | **Sequence** |
| β-actin | 5'-CATGTACGTTGCTATCCAGGC-3' (forward)  5'-CTCCTTAATGTCACGCACGAT-3' (reverse) |
| GAPDH | 5’-TTGCCATCAATGACCCCTTCA-3’ (forward)  5’-CGCCCCACTTGATTTTGGA-3’ (reverse) |
| BRAF | 5’-AAAATAGGTGATTTTGGTCTAGCTACAGA-3’ (forward)  5’-GACAACTGTTCAAACTGATGG-3’ (reverse) |
| ERK-1 | 5’-CGCTTCCGCCATGAGAATGTC-3’ (forward)  5’-CAGGTCAGTCTCCATCAGGTCCTG-3’ (reverse) |
| ERK2 | 5’-CGTGTTGCAGATCCAGACCATGAT-3 (forward)  5’-TGGACTTGGTGTAGCCCTTGGAA-3’ (reverse) |
| PI3K | 5’-ATGGGGATGATTTACGGCAAGATA-3’ (forward)  5’-TTTCGCACCACCTCAATAAGTC-3’ (reverse) |
| AKT | 5’-CCCCCGAGGTGCTGGAGGACAAT-3’ (forward)  5’-AAGGGCAGGCGACCGCACATCAT-3’ (reverse) |
| m-TOR | 5’-GCTTGATTTGGTTCCCAGGACAGT-3’ (forward)  5’-GTGCTGAGTTTGCTGTACCCATGT-3’ (reverse) |

Primers from Ludwig Biotecnologia, Brazil. Abbreviations: GAPDH, glyceraldehyde 3-phosphate dehydrogenase.


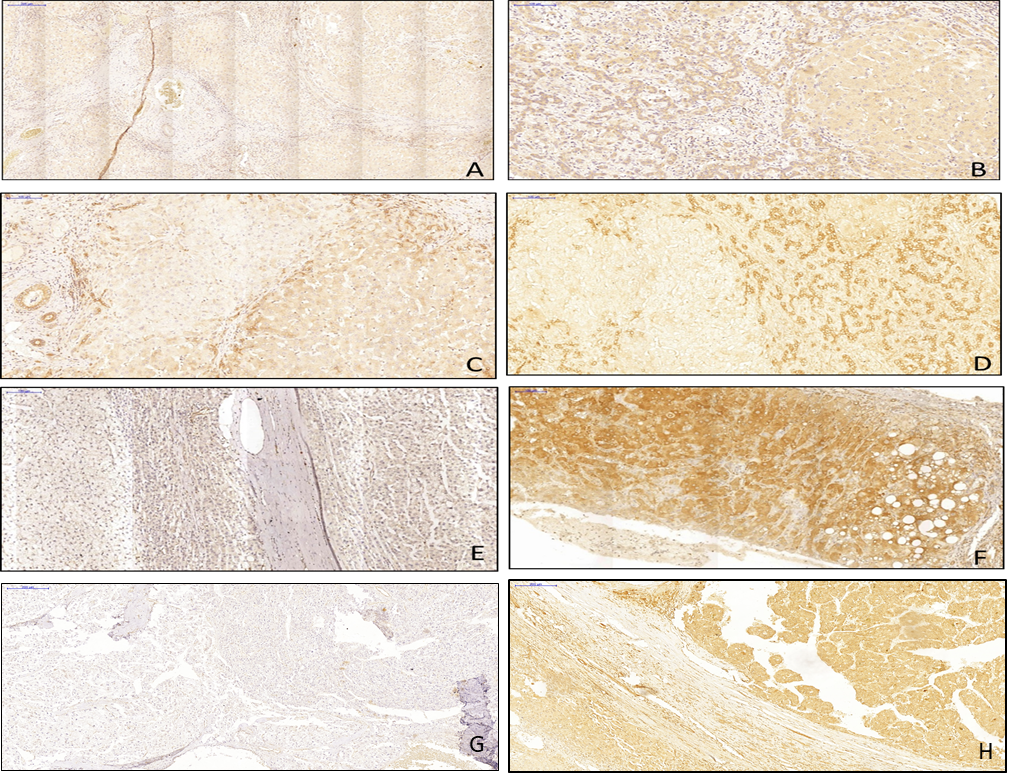


**WEAK EXPRESSION IN TUMOR AND CIRRHOSIS**

**STRONG EXPRESSION IN TUMOR, WEAK IN CIRRHOSIS**

**Figure S1 (A-H):** **Representative staining patterns**. Immunohistochemistry is expressed as a final score summarizing the result of intensity and extent of staining in tumor and adjacent cirrhosis. Left columns: weak expression in tumor and adjacent cirrhosis; right columns: strong expression in tumor, weak expression in adjacent cirrhosis. A, B: KRAS; C, D: B-RAF; E, F: MEK-1; G, H: ERK 1/2

**WEAK EXPRESSION IN TUMOR AND CIRRHOSIS**

**STRONG EXPRESSION IN TUMOR, WEAK IN CIRRHOSIS**


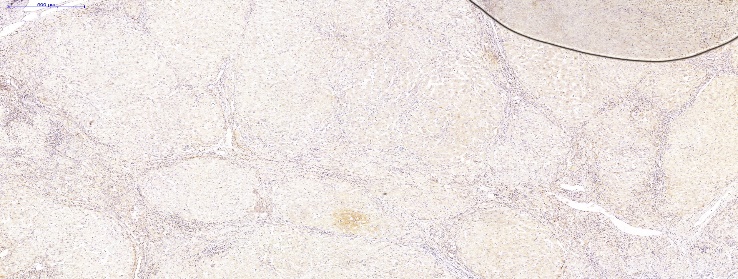

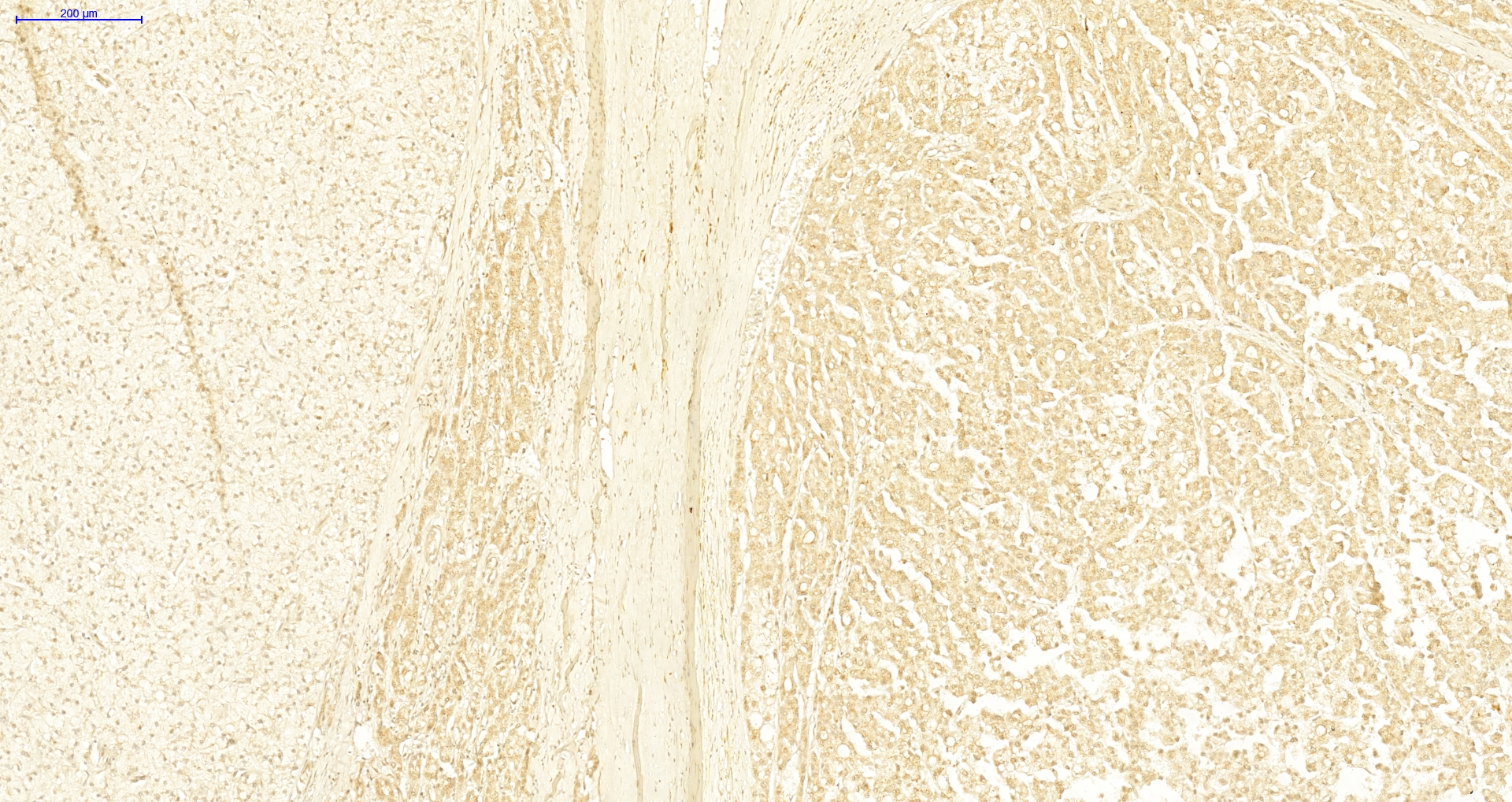

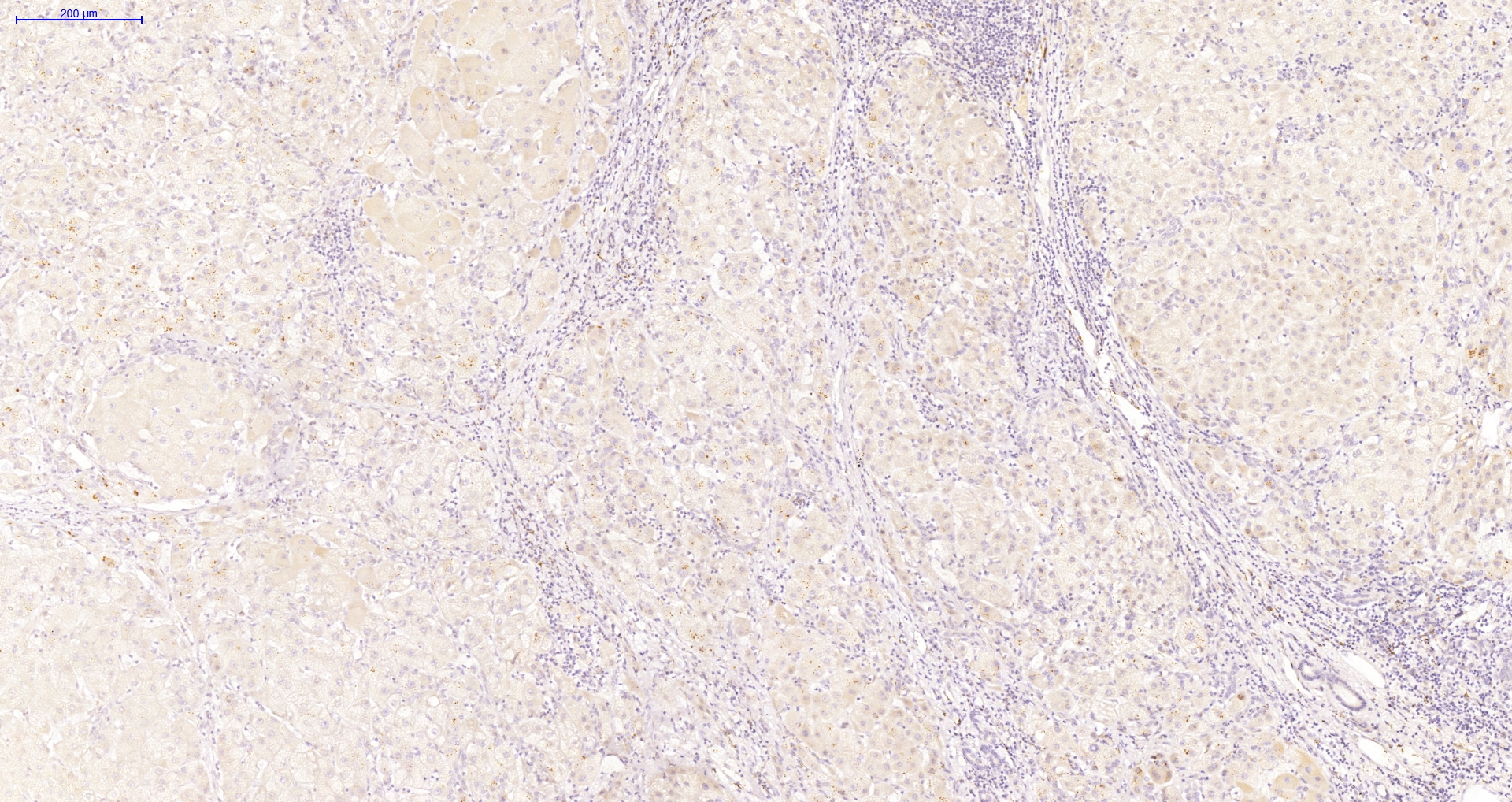

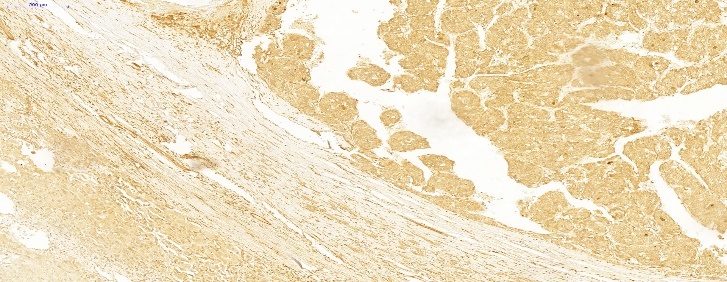

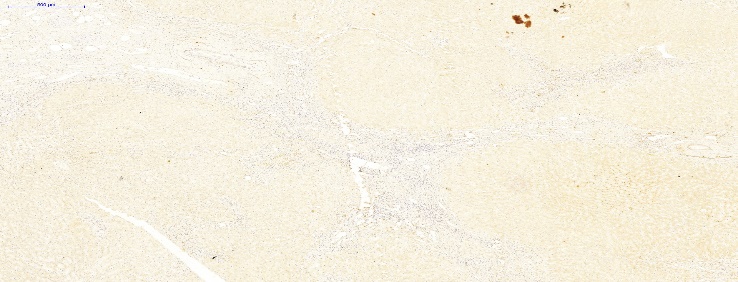

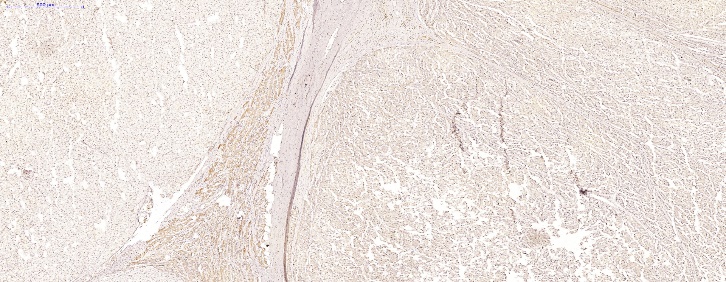


A

B

C

D

E

F

**WEAK EXPRESSION IN TUMOR AND CIRRHOSIS**

**STRONG EXPRESSION IN TUMOR, WEAK IN CIRRHOSIS**

**Figure S2 (A-F):** **Representative staining patterns**. Immunohistochemistry is expressed as a final score summarizing the result of intensity and extent of staining in tumor and adjacent cirrhosis. Left columns: weak expression in tumor and adjacent cirrhosis; right columns: strong expression in tumor, weak expression in adjacent cirrhosis. A, B: PI3K; C, D: AKT; E, F: mTOR
